# Functional, physicochemical properties of sodium carbonate-soluble polysaccharides from the bulbs and foliage leaves of yellow and red onion

**DOI:** 10.1038/s41598-024-77936-z

**Published:** 2024-11-01

**Authors:** Magdalena Marciniak, Jolanta Cieśla, Monika Szymańska-Chargot, Justyna Cybulska, Artur Zdunek

**Affiliations:** Institute of Agrophysics, Polish Academy of Sciences, Doświadczalna 4, Lublin, 20-290 Poland

**Keywords:** Gelling ability, Oil holding, Common onion pectin, Surface activity, Water holding, Polysaccharides, Self-assembly

## Abstract

The oil and water holding capacities, surface activity, and gelling ability of sodium carbonate-soluble pectin (NSP) extracted from the cell wall of bulb and foliage leaves of yellow and red onion (*Allium cepa* L.) were investigated and compared with those of commercial citrus pectin. Pectin chemical composition and properties its aqueous dispersions (e.g. the viscosity, pH) were studied. Homogalacturonan was the main component of the low-methoxylated pectin, with a small amount of rhamnogalacturonan I (more branched in the bulb pectin). Both the oil (35–41 g/g_d.m_.) and water (20 g/g_d.m_) holding capacities of NSP were higher than citrus pectin (1 and 17 g/g_d.m_, respectively). The surface activity of NSP was comparable (foliage leaves; surface tension (γ) decrease to 62 mN/m) or higher (bulb; γ decrease to 56 mN/m) than commercial pectin. The ability of NSP, especially extracted from the bulb, to form larger structures with increasing viscosity and neutralizing the negative surface charge, was significantly higher than that of citrus pectin. Therefore, NSP of bulb and foliage leaves may be useful as a carrier of oil- or water-soluble substances, a surface active agent, texturizer and gelling agent in the food, pharmaceutical, cosmetic and agricultural branches of industry.

**Supplementary Information:**

The online version contains supplementary material available at 10.1038/s41598-024-77936-z.

## Introduction

The common onion (*Allium cepa* L., the Latin “*cepa”* means “onion”) is a popular vegetable, valued mainly for its pro-health properties. The presence of bioactive substances (e.g. phenolic compounds) makes this plant useful for the food, pharmaceutical, and cosmetic industries^1,2^. The onion bulb is rich in polysaccharides. As it was shown by Ng et al., the cold alcohol-insoluble cell wall material of onion bulb (the yield of about 4% of fresh weight) is composed of over 80% polysaccharides^3^. The dominant polysaccharide is pectin, mainly homogalacturonan (HG), followed by cellulose and hemicelluloses, mainly xyloglucan. However, these polysaccharides’ structure and chemical composition are related to the onion tissues^3–6^. Some fractions of the onion bulb polysaccharides reveal antioxidative activity as the functional, biochemical property^7,8^.

The application of the sequence of solvents in the process of polysaccharides extraction allows the separation of the fractions with various properties and functions. Pectic polysaccharides, in order from the weakest to the strongest bonded in the cell wall, can be extracted using, e.g. the water, imidazole, cyclohexane-trans-1,2-diamine-NNN’N’ tetra acetate, and finally sodium carbonate solution. The next application of differentially concentrated potassium hydroxide solutions results in the obtaining hemicelluloses fraction^3^. Sodium carbonate – soluble pectin (NSP), also called diluted alkali – soluble pectin (DASP), has been in the area of scientists’ interest due to both its impact on the quality of fruits and vegetables and the ability to macromolecular network formation in the solution. Scientific works have been mainly focused on the relationship between the structure and/or content of this pectin and the fruit ripening (e.g. tomatoes^9^), the cell wall breakdown, and the change in the texture of fruit and vegetable during the post-harvest storage (e.g. asparagus^10^, and apples^11^), and in the processing of fruit and vegetables (e.g. pickling^12^, pressure-cooking^13^, and blanching^14^). Results obtained by Lecain et al. showed that the process of softening of onion bulb during the pressure-cooking was connected with a decrease in the amount of NSP^13^. On the molecular level, it was observed, that the NSP extracted from the pear fruit^15^ and carrot^16^, were able to self-organize on mica. The density of a network may affect the texture and firmness of the fruit. It was postulated, that the specific structures formed on mica were connect with the presence of arabinose, galactose, and rhamnose in the pectin chain^17^. In further research, it was found, that rhamnose was responsible for the spatial structure of “kinked rods” and the pectin ability for gelling^18^. Pectin influences the viscoelastic properties of the onion cell wall^19^. This biopolymer is composed of the smooth region formed by α-1,4 linked d-galacturonic acid residues with different methyl-esterification degree, i.e. homogalacturonan (HG), and the hairy (ramified) regions with the arabinan, arabinogalactan, and xylogalacturonan side chains, i.e. rhamnogalacturonan I (RG I), and rhamnogalacturonan II (RG II) characterized by a complicated structure^20,21^. In the case of functional, physicochemical properties of the onion bulb polysaccharides, it was shown by Zhu et al.^22^, that fat binding, foaming, moisture retention, emulsifying ability and viscosity are fraction-specific, and in some cases, concentration-dependent. The NSP, especially this extracted from the foliage leaves of onion, has not been studied.

The commercially available pectin is produced primarily from citrus peels through the acidic hydrolysis process. Low methoxylated pectin (the degree of methyl esterification (DM) < 50%) is often amidated to increase its rheological properties and decrease the amount of multivalent cations needed for gelation^21^. Citrus pectin is used mainly by the food sector, as a texturizer, stabilizer, gelling agent, emulsifier, and component of packaging and 3D printed food, but also in the medicine – as a biomaterial, and in the pharmacy, e.g. as an encapsulating agent^21,23^. The ability to gel formation by pectin is also of interest to the agrochemical industry looking for the natural, biodegradable carriers of agricultural nutrients^24^.

Considering the current need to reduce the waste of fruits and vegetables, the demand of various branches of industry for pectic polysaccharides, the interesting properties of NSP of different fruits and vegetables, huge global production of onion, the known functional properties of some fractions of onion bulb polysaccharides, and the lack of information on the structure and physicochemical properties of NSP of onion foliage leaves, the research presented below was undertaken.

Studies aimed to characterize the chemical composition and the functional physicochemical properties of NSP sequentially extracted from the bulbs and foliage leaves of yellow (*Allium cepa* L. var Wolska) and red (*Allium cepa* L. var Karmen) onions, and compare them with the properties of commercial citrus pectin. Two onion varieties were selected to evaluate the impact of the variety and organ of the plant on the properties of NSP.

To the best of our knowledge, this is the first such complementary study of the physicochemical properties of NSP pectin isolated from onion bulbs and foliage leaves which opens the further research on industrial applications.

## Results and discussion

### The structure of the NSP fraction

The first stage of the investigation was determination of the chemical composition and structure of NSP fraction extracted from the onion cell wall and commercially available citrus pectin. This information was crucial to confirm the pectic character of polysaccharides, and next to find their structure – functionality relationship. Studied polysaccharides were labelled as: NSP YO B (NSP of yellow onion bulb), NSP YO L (NSP of yellow onion foliage leaves), NSP RO B (NSP of red onion bulb), NSP RO L (NSP of red onion foliage leaves), and LMA P (low-methyl-esterified amidated citrus pectin). Their composition determined using high-performance liquid chromatography (HPLC) is summarized in Table 1..

**Table 1.** Monosaccharides and uronic acids content (mol %) in NSP and LMA P.

	The component	The sample				
		NSP YO B	NSP YO L	NSP RO B	NSP RO L	LMA P
The pectin component content (mol %)	Ara	4.8 ± 0.1^a^	5.5 ± 0.5^a^	2.4 ± 0.3^b^	4.9 ± 0.1^a^	3.1 ± 0.3^b^
	Fuc	2.8 ± 0.3^b^	3.3 ± 0.1^a^	0.4 ± 0.1^cd^	0.7 ± 0.1^c^	0.0 ± 0.0^d^
	Gal	18.7 ± 1.0^a^	12.0 ± 1.0^c^	11.2 ± 0.5^c^	8.3 ± 0.3^d^	14.9 ± 0.1^b^
	Glc	0.5 ± 0.1^c^	1.7 ± 0.3^b^	3.2 ± 0.2^a^	3.1 ± 0.3^a^	0.0 ± 0.0^d^
	Man	0.8 ± 0.1^c^	0.6 ± 0.1^c^	3.6 ± 0.1^a^	2.9 ± 0.5^b^	0.0 ± 0.0^d^
	Rha	3.7 ± 0.3^b^	3.5 ± 0.3^b^	3.3 ± 0.3^b^	3.1 ± 0.6^b^	5.5 ± 0.1^a^
	Xyl	0.2 ± 0.0^b^	0.1 ± 0.1^b^	0.3 ± 0.1^b^	2.4 ± 0.2^a^	0.2 ± 0.0^b^
	GalA	67.4 ± 1.2^b^	72.4 ± 2.3^a^	74.9 ± 1.6^a^	73.9 ± 2.0^a^	74.8 ± 0.3^a^
	GlcA	1.1 ± 0.3^ab^	0.9 ± 0.2^b^	0.7 ± 0.1^b^	0.7 ± 0.1^b^	1.5 ± 0.1^a^
	HG	63.7 ± 1.1^b^	68.9 ± 2.5^a^	71.5 ± 1.9^a^	70.8 ± 2.6^a^	69.3 ± 0.3^a^
	UA	68.5 ± 1.2^b^	73.3 ± 2.1^a^	75.6 ± 1.5^a^	74.6 ± 2.0^a^	76.3 ± 0.4^a^
Ratios	Rha/UA	0.05 ± 0.01^b^	0.04 ± 0.01^b^	0.04 ± 0.01^b^	0.04 ± 0.01^b^	0.07 ± 0.0^a^
	UA/(Gal+Ara)	2.9 ± 0.2^c^	4.2 ± 0.5^b^	5.6 ± 0.4^a^	5.6 ± 0.3^a^	4.2 ± 0.1^b^
	(Gal+Ara)/Rha	6.4 ± 0.8^a^	5.0 ± 0.4^b^	4.1 ± 0.2^bc^	4.4 ± 0.8^bc^	3.3 ± 0.1^c^

Ara – arabinose, Fuc – fucose, Gal – galactose, Glc – glucose, Man –mannose, Rha – rhamnose, Xyl – xylose, GalA – galacturonic acid, GlcA –glucuronic acid, HG – homogalacturonan, UA – the total content of uronic acids; the results are expressed as the average of three values ± standard deviation; various letters mean different results in the rows based on the two-way ANOVA and post-hoc Tukey’s test (p < 0.05)

The content of GalA in all polysaccharides was higher than 65 mol %, confirming their pectic character. The UA and HG content was the same in the red onion NSP and LMA P, and it was higher than in the yellow onion NSP. The smooth HG was the main component of NSP and LMA P. Considering the Rha/UA, the share of hairy RG I in the NSP structure was lower than in LMA P. Analysis of the UA/(Gal + Ara) revealed differences between NSP of yellow (more branched) and red onion. Moreover, the NSP of leaves was less branched than the bulbs. The average length of the RG I side chains (based on (Gal + Ara)/Rha) increased from LMA P, through the red onion NSP to the yellow one. The content of Ara and Fuc increased starting from LMA P, through the red onion NSP to the yellow one. The content of Ara, Fuc, Glc, and Xyl was higher in the NSP of leaves than the bulbs, oppositely to the Gal and Man content. The content of Gal in the yellow onion NSP and LMA P was the same, and it was higher than in the red onion NSP (the richest in Xyl). The Glc and Man were not detected in LMA P, and their content in the red onion NSP was higher than in the yellow one. The data obtained for the onion bulb NSP (Table 1.) corresponded to the results presented by other scientists. According to Ng et al.^3^, Lecain et al.^13^, and O’Donoghue et al.^25^, the UA, Gal, and Ara were the main components of NSP. This onion bulb pectin was highly branched by galactans and arabinogalactans^3,26^. The published data related to the NSP of onion foliage leaves has not been found.

Physical and physicochemical properties of pectin macromolecules as well as the presence of additional substances can affect pectin functionality. Therefore, NSP and LMA P were characterized in terms of molecular weight, hydrodynamic diameter, and surface electrical charge (laser light scattering), methyl-esterification and amidation degrees (FTIR), and the content of proteins and phenolic compounds (UV-vis). Results are summarized in Table 2.

**Table 2:** The chemical and physicochemical characteristics of the onion NSP and LMA P.

The characteristic	The sample				
	NSP YO B	NSP YO L	NSP RO B	NSP RO L	LMA *P*
MW (kDa)	5,260 ± 442^ab^	3,688 ± 716^bc^	5,591 ± 858^a^	1,085 ± 51^d^	2,385 ± 547^cd^
A_2_ (µl·cmol/g^2^)	-13 ± 1^ab^	-18 ± 3^b^	-10 ± 1^a^	-53 ± 1^c^	-18 ± 4^b^
DM (%)	11 ± 2^b^	12 ± 1^b^	9 ± 1^b^	11 ± 1^b^	25 ± 0^a^
DA (%)	-	-	-	-	30 ± 1
Z_ave,0_ (nm)	827 ± 59^a^	842 ± 70^a^	699 ± 37^bc^	587 ± 20^c^	761 ± 32^ab^
PdI_0_·10^2^	59 ± 1^b^	60 ± 6^b^	72 ± 6^a^	60 ± 1^b^	29 ± 2^c^
Q_0_ (µC/m^2^)	-147 ± 6^a^	-136 ± 8^a^	-138 ± 5^a^	-146 ± 10^a^	-140 ± 11^a^
NaClSprotein (µg/g_d.m._)	290 ± 55^d^	594 ± 78^c^	379 ± 38^d^	1,694 ± 8^b^	2,475 ± 63^a^
TPC (µg/g_d.m._)	220 ± 32^b^	241 ± 32^ab^	306 ± 32^a^	301 ± 31^ab^	69 ± 10^c^

MW – weight averaged molecular mass, A_2_ – second virial coefficient, DM – methyl-esterification degree, DA – amidation degree, Z_ave,0_ – the mean hydrodynamic diameter of macromolecules in the aqueous dispersion at a given polydispersity index (PdI_0_), Q_0_ – the net surface electrical charge of macromolecule, NaClS protein – the protein soluble in physiological salt solution, TPC – total phenolic content; the results are expressed as the average of three values ± standard deviation; various letters mean different results in the rows based on the two-way ANOVA and post-hoc Tukey’s test (*p* < 0.05).

**Fig. 1:**
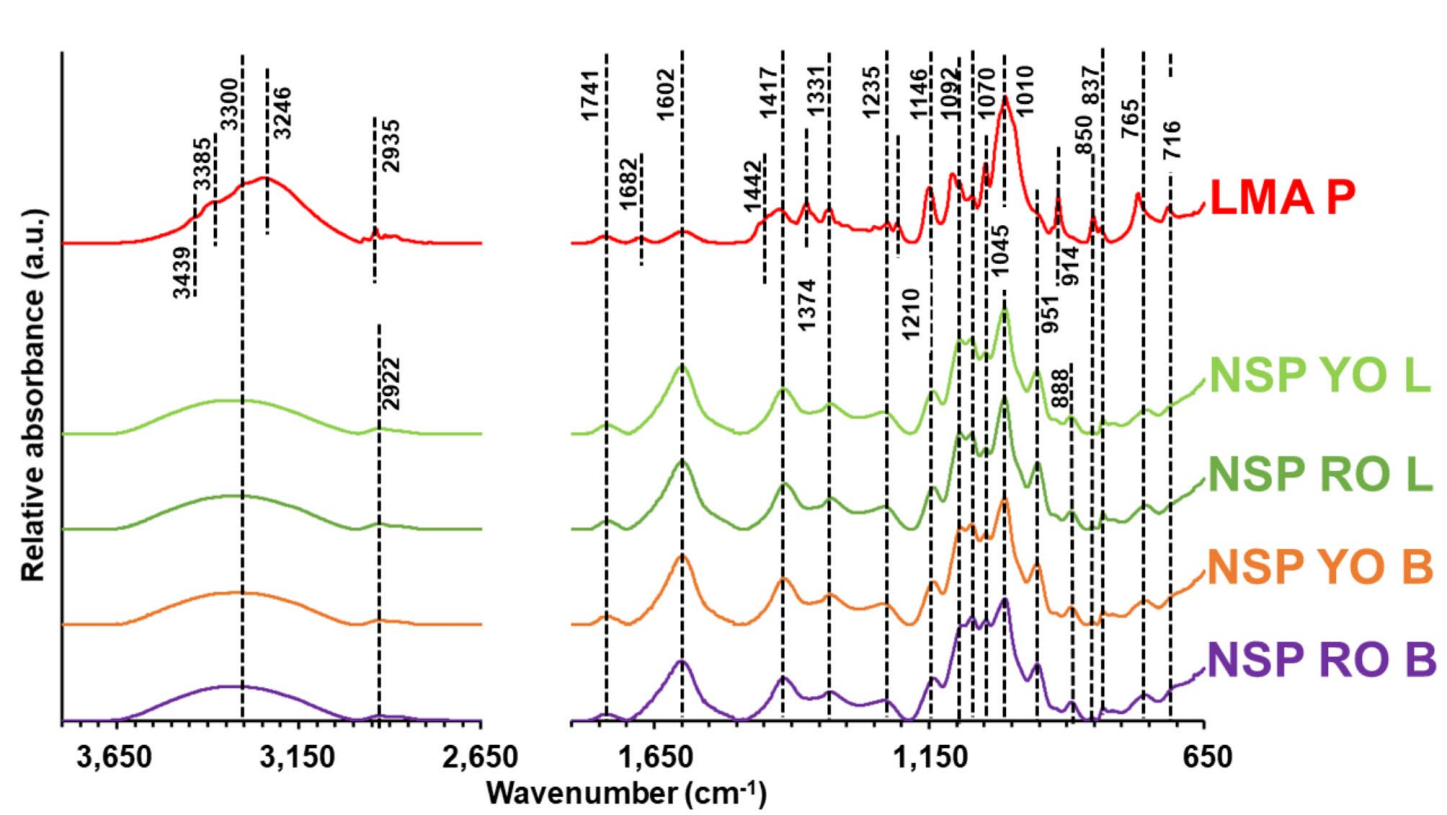
The FT-IR spectra of the onion NSP and LMA P.

The MW of NSP was higher than LMA P. Moreover, MW of the onion leaves NSP was lower than that of bulbs. The negative values of A_2_ pointed out the tendency of pectin macromolecules to aggregate in the water^27^. Hydrodynamic diameter of macromolecules in the ultrapure water increased in the following order: red onion NSP < LMA P < yellow onion NSP, and was not affected by the onion organ. LMA P was monodisperse. The red onion bulb NSP was the most heterogeneous in the particle size. The net surface electrical charge of pectin in the ultrapure water was negative. It was consistent with the data obtained for the apple^28^ and pear^29^ NSP, as a consequence of the presence of COO^−^ groups in the pectin. The MW of LMA P and the onion NSP was higher than that of the pear^30^, apple, and carrot^31^ NSP. Each pectin was low-methyl-esterified. DM of LMA P was about three times higher than that of onion NSP. The DM of the onion NSP was higher than that of the pear^30^, apple, and carrot^31^ NSP, and lower than the onion hulls NSP^32^. The analysis of FTIR spectra (Fig. 1.) showed that only LMA P was amidated. The highest amount of proteins was determined in LMA P, but that could be connected with its amidation. NSP from onion leaves was richer in proteins than that from bulbs. Among the onion leaves NSP, the highest amount was determined for red onion. The determined proteins were probably ionically or covalently linked with pectin^33^. Their presence may be important for agricultural NSP application due to the possible antifungal activity^34^. However, further studies are needed in the aspect of such bioactivity of onion NSP. The presence of proteins in other sequentially extracted polysaccharides of red onion bulb was shown also by Zhu et al.^22^. The LMA P was 3–4 times poorer in the phenolic compounds than the onion NSP. The lowest TPC was in NSP YO B. The analysed phenolic compounds were probably non-covalently bound with pectin^35^. Their presence may be important for the onion NSP application in the food, pharmaceutical, cosmetics and agricultural branches of industry due to the antioxidant, antifungal and antimicrobial activity^1,2,7,8^. Therefore, this can be an impulse for further studies on the bioactivity of the onion NSP. The analysis of correlation (Supplementary Table S1) revealed that the NSP containing lower amounts of proteins was characterized by higher MW and Z_ave,0_. The higher NSP branching resulted in a higher Z_ave,0_. Polydispersity increased with an increasing TPC. TPC positively correlated with the HG and UA contents, and the UA/(Gal + Ara), while negatively – with the (Gal + Ara)/Rha. This suggested that phenolic components were non-covalently bound to the HG^36^. Their release into the water resulted in an increase in the particle size heterogeneity.

Confirmation of the structure and composition of NSP and LMAP was sought by analysing their FT-IR spectra (Fig. 1). In the wavenumber range from 4000 to 2500 cm^−1^^37^, the bands connected with the NH stretching vibration of amide (3439 and 3385 cm^−1^), intermolecularly bonded OH stretching (3246 cm^−1^)^38^, and the CH stretching of amide (2935 cm^−1^;^39^) were found for LMA P. For each pectin the band at 3300 cm^−1^, referring to OH stretching vibration^38^, was visible. For the NSP, the band at 2922 cm^−1^, connected with the CH stretching vibrations^37^ was present.

In the region of the “double bonds stretching”^37^, the bands at 1741 and 1602 cm^−1^, corresponding to the CO stretching in alkyl ester and the COO^−^ asymmetric stretching of the ester group of pectin, respectively, were visible for each pectin. Moreover, the CN stretching of amide I reflected by the band at 1682 cm^−1^^40^, was detected on the LMA P spectrum. In the “local symmetry” region, the bands connected with the following vibrations were identified: the deformation NH vibration of amide II (1442 cm^−1^;^40^) and amide III (1210 cm^−1^;^38^), and the CH_2_ deformation and CC perpendicular stretching in pectin (1374 cm^−1^;^41^), visible for LMA P, the COO^−^ symmetric stretching of the pectin ester group (1417 cm^−1^;^40^), the CH deformation of pectin ring (1331 cm^−1^;^40,41^), the CO stretching in pectin^40^ as well as the interaction between C-N stretching and C-N-H in-plane bending in protein (1235 cm^−1^)^42^ detected for each pectin. In “a fingerprint” (1200–800 cm^−1^) region, reflecting the presence and configuration of glycosidic linkage in polysaccharides^37^, the following group vibrations were visible: the COC glycosidic bonds of HG (1146 cm^−1^;^40,41^), the CO and CC stretching in pectin ring (1092 cm^−1^;^40^), RG I and galactan (1070 cm^−1^;^41^), the CO and CC stretching and the OCH deformation parallel in pectin (1045 cm^−1^;^41^), the C2-C3, C2-O2 and C1-O1 stretching (1010 cm^−1^) and the CO bending (951 cm^−1^) in pectin^40^, the CO stretching methylene acetal (914 cm^−1^;^38^) visible especially for LMA P, and the band at 888 cm^−1^^40^ probably related to the β-galactopyranose residue vibration^43^. The COC asymmetric stretching of the glycosidic linkage of acidic pectin (850 cm^−1^;^42^), and the pectin ring vibration (837 cm^−1^;^40^) were also visible. In the last one, the “skeletal region” of FT-IR spectra (the wavelength lower than 800 cm^−1^;^37^), only two bands (765 and 716 cm^−1^), probably connected with the CH out-of-plane bending of a phenyl group^38^ were visible. Therefore, the FT-IR spectra revealed the bands that are characteristic of the pectin. The main difference between the NSP and LMA P was the presence of amide groups in the commercial citrus pectin. The occurrence of proteins and polyphenols in the pectin was also reflected in the spectra. However, their low content (Table 2) resulted in low band intensity in the spectra.

## The physicochemical, functional properties of onion NSP

### The oil/water holding and the surface properties

**Fig. 2:**
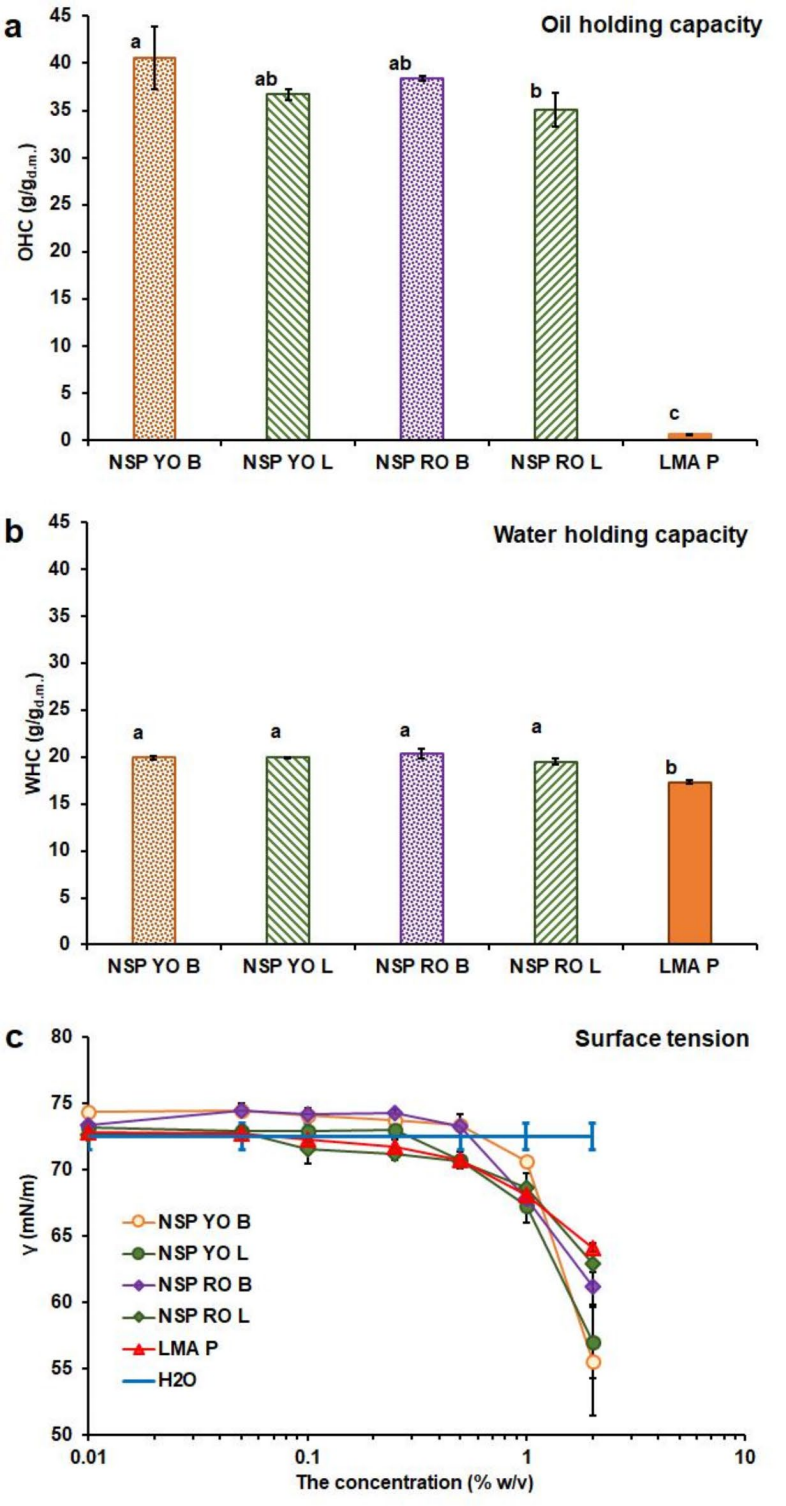
**a** The OHC, **b** the WHC, and **c** the effect of pectin concentration on the surface tension (γ) of the aqueous dispersion at 20 °C; the results are expressed as the average of three values ± standard deviation (the bars); various letters mean significantly (*p* < 0.05) different results.

The ability of onion NSP to hold both the oil and the water (Fig. 2a and b) was significantly higher than that of LMA P. The OHC (oil holding capacity) of bulb NSP was slightly higher than that of leaves. The NSP OHC was many times higher than onion bulb polysaccharides obtained using extraction with other solvents^22^. This can be a result of differences in the spatial structure of macromolecules and the network formed by them^22^. Both the OHC and WHC increased with an increase in MW (Supplementary Table S1). This confirmed that liquids were absorbed in the volume of the structure formed by NSP molecules^22^. Therefore, the onion NSP may be useful not only for food, drugs, and cosmetics formulations containing oil and oil-soluble bioactive components, but also in the context of its ability to sorb oil contaminants, e.g. in the soil. WHC (water holding capacity) and hydrogel formation also can find application, e.g. in the production of burn dressing, cosmetic masks or agro-materials for water retaining.

The surface activity of aqueous dispersions of pectin was evaluated by determination of surface tension (γ) (Fig. 2c). For each pectin at a concentration higher than 0.5% w/v, a significant decrease in the γ occurred (Supplementary Table S2.). The yellow onion NSP was more effective in lowering γ than the red onion NSP. Higher surface activity was determined for the NSP with a shortened HG chain and more branched structure (Supplementary Table S1). This aspect was consistent with other published information on the effect of MW, DM, chemical structure of backbone, and branching of pectin, and the presence of other substances on the pectin interfacial properties^44,45^. The surface activity of the NSP may be important for potential application in the emulsion formulation.

## The NSP structure-forming abilities

**Fig. 3:**
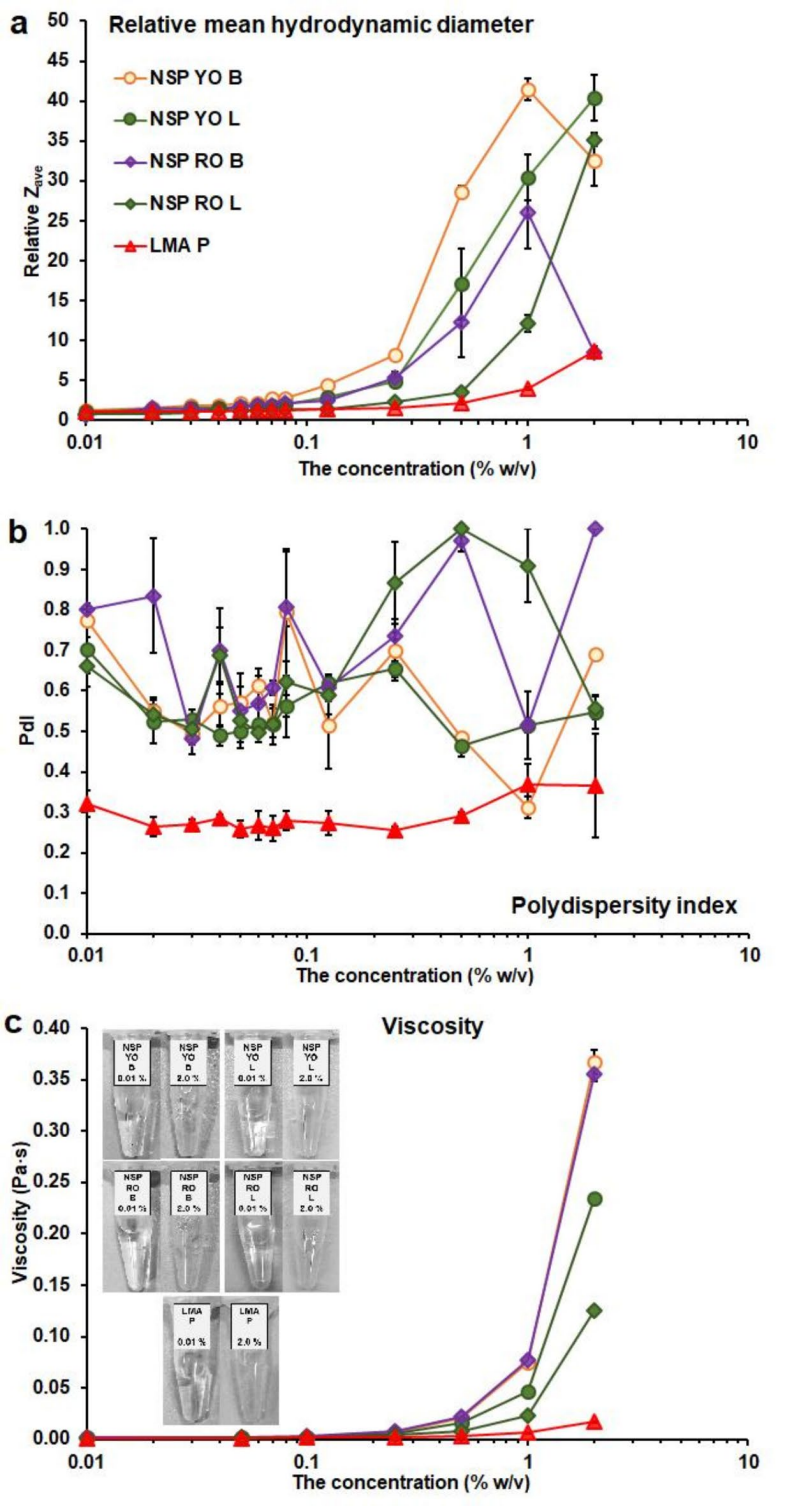
Dependence of **a** – relative mean hydrodynamic diameter (Relative Z_ave_), **b** – polydispersity index (PdI), and **c** – viscosity measured at a shear rate of 200 s^−1^ on the pectin concentration in the aqueous dispersion at 20 °C; the photos of the samples at the lowest and the highest concentration is shown in Fig. 3c; the results are expressed as the average of three values ± standard deviation (the bars).

The ability of pectin macromolecules to connect with each other and create larger structures was studied using laser light scattering. The relative mean hydrodynamic diameter (Relative Z_ave_) of the pectin particles dispersed in the ultrapure MilliQ water was obtained by dividing the Z_ave_ determined at a given pectin concentration by Z_ave,0_ of LMA P. Above a concentration of 0.125% w/v (Supplementary Table S3), a significant increase in Relative Z_ave_ of NSP YO B occurred (Fig. 3a). For NSP YO L and NSP RO B (0.25% w/v), NSP RO L (0.50% w/v), and LMA P (1% w/v) a higher concentration was needed. Generally (Supplementary Table S9), molecules with the higher content of HG, UA, protein, and phenolic component, but the lower MW, Zave,_0_ and branching, tended to self-organize at the higher concentration of aqueous dispersion. An increase in Relative Z_ave_ of onion NSP was much more intense compared to LMA P. The bulb NSP revealed a better ability to Relative Z_ave_ increase than that of leaves. The observed increase in the particle size was connected with the self-organization and three-dimensional network formation by the pectin macromolecules in the concentrated systems^46^. Such results were previously denoted for the apple^28^, and pear^29,30^ NSP, during a gel formation. The decrease in Relative Z_ave_ denoted for the bulb NSP (2% w/v), was connected with the network formation by the macromolecules^46^. The onion NSP showed a better ability to aggregate than the commercially available citrus LMA P. The gelling process of different pectic fractions of onion polysaccharides will be shown more precisely in further work. Polydispersity Index (PdI) (Fig. 3 ) characterises the pectin dispersion in terms of the diversity of particle shapes and sizes. The LMA P was very homogeneous (PdI 0.3) in the whole range of concentration.

The PdI of onion NSP was higher than 0.5. For the red onion NSP the PdI was changeable, while for the yellow onion NSP, it slightly decreased with an increase in concentration (Supplementary Table S4). The higher PdI was determined for NSP with the lower MW, Z_ave,0_, and branching, and the higher content of HG, UA, protein, and phenolic components (Supplementary Table S9). The high PdI of NSP was a reason that the relative, not an exact values of Z_ave_ were considered. An increase in the viscosity of polysaccharide suspension to infinity is an indicator of the initiation of a network formation^47^. The relationship between the viscosity of the pectin dispersions and the concentration is shown in Fig. 3c. The viscosity of NSP was higher than LMA P. Among NSPs, the yellow onion NSP was higher than the red one. Moreover, the viscosity of the bulbs’ NSP was higher than that of leaves. Different concentrations were needed to observe a significant increase in viscosity. The lowest (0.25% w/v) was for the NSP of yellow onion and the bulb of red one, the higher (0.50% w/v) for the NSP of red onion leaves, and the highest (1% w/v) for LMA P (Supplementary Table S5). This concentration was higher for the pectin with the higher content of protein and the lower MW and Z_ave,0_ (Supplementary Table S9). The obtained results pointed out the ability of pectin to stick and its texture-forming properties, those were higher for the onion NSP than commercial LMA P, and the highest – for the onion bulb NSP. It was also shown by Zhu et al., that the polysaccharides of red onion bulbs had such properties. However, there was no NSP, and the best texturizing properties were revealed by a calcium chelator-soluble fraction^48^.

**Fig. 4:**
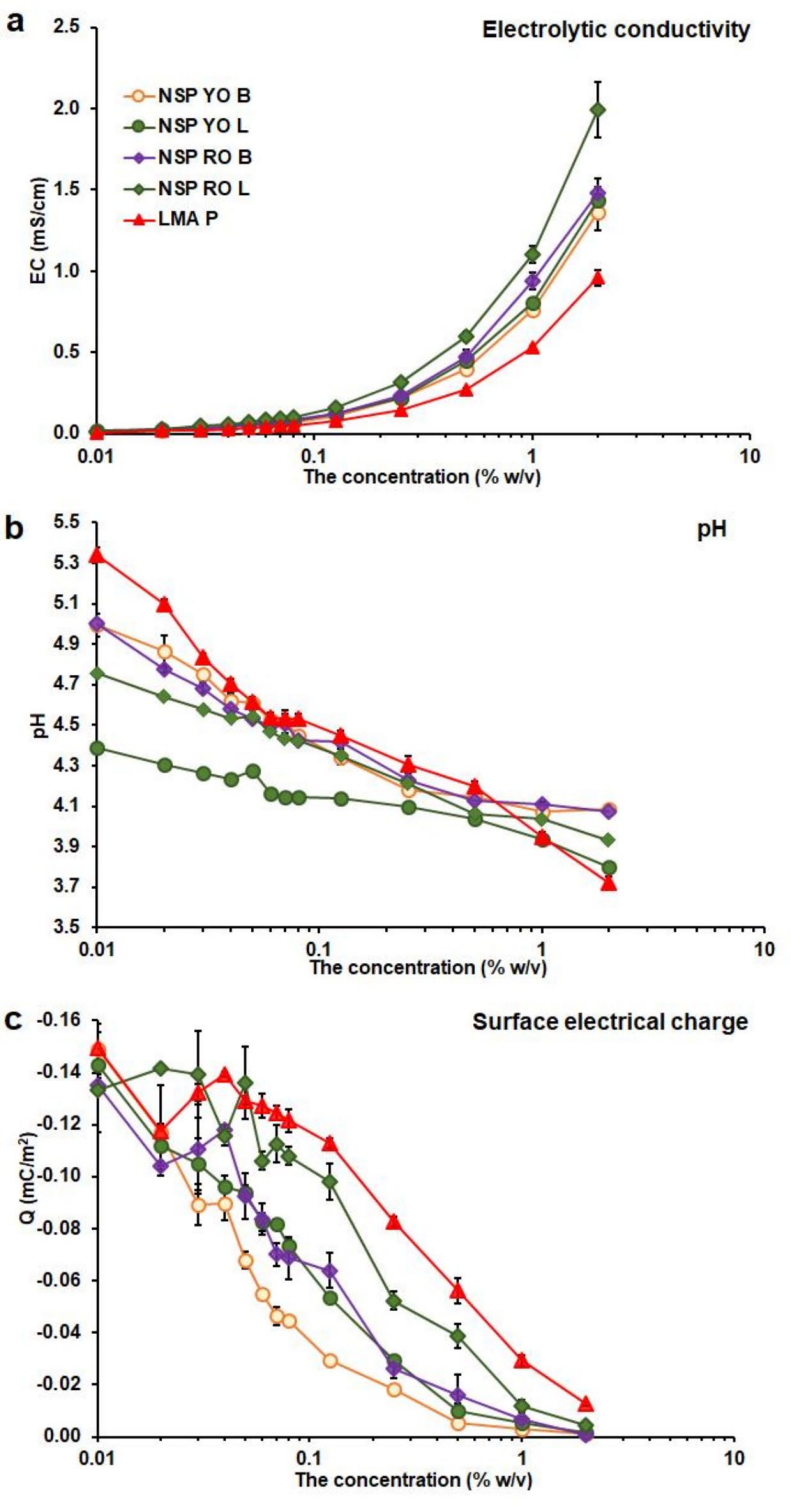
An effect of pectin concentration on **a** the electrolytic conductivity (EC), **b** pH of aqueous dispersion, and **c** net surface electrical charge (Q) of dispersed particles; the results are expressed as the average of three values ± standard deviation (the bars).

The structural changes in pectin dispersion were connected with the ionic character of macromolecules. These dispersions were rich in ionic components that were the electrical charge carriers (Fig. 4a). The electrolytic conductivity (EC) of NSP dispersion was higher than LMA P. The EC of leaves NSP was higher than that of bulbs. A significant increase in EC occurred at a concentration higher than 0.125% w/v (0.08% w/v for red onion leaves NSP) (Supplementary Table S6). EC positively correlated with the content of HG, UA, protein, and phenolic compounds. However, the high EC did not promote the self-organization of macromolecules due to the higher concentration needed to increase Relative Z_ave_ and viscosity (Supplementary Table S9). The results of pH measurements (Fig. 4b) of aqueous dispersions confirmed the acidic character of pectin. The lowest pH was for the yellow onion NSP, higher for red onion NSP, and the highest for LMA P. The pH of the bulb NSP dispersion was higher than that of leaves. As pectin concentration increased, pH decreased due to the dissociation of acidic functional groups, mainly carboxyl groups of polygalacturonic acid^28^.

In the case of NSPs YO B, beginning from 0.50% w/v, an increase in the concentration did not cause a pH decrease (Supplementary Table S7). This may be due to the intense formation of intermolecular hydrogen bonds^29^. The lowering pH with an increase in the NSP concentration in water was also observed for the apple^28^ and the pear^29^ NSP. It was shown, that the hydrogen ion binding increased with increasing pectin concentration^28,29^. Simultaneously with an increase in pectin concentration, the intermolecular distance was shortened and bigger structures were formed due to intermolecular hydrogen bridges^29^. As a result of that, the absolute value of the net surface electrical charge (Q) of dispersed pectin particles decreased (Fig. 4c). This process was the most rapid in the case of NSP YO B. In concentrated NSP dispersions, the Q was constant and close to 0 (Supplementary Table S8). Commercial LMA P had the highest absolute value of Q throughout the experiment. The lower absolute value of Q in the concentrated system was for NSP with higher MW and Z_ave,0_, and lower PdI and EC. The role of MW in pectin functionality was also stressed by Neckebroeck et al. for onion^44^, and by Wang et al. for citrus pectin^49^. A decrease in the absolute value of Q with an increase in pectin concentration was previously shown for the pear NSP. The final Q (at 2% w/v) was almost neutral^29^. Low-methoxylated NSP can form a weak gel in acidic conditions when the dissociation of carboxylic groups is low and the intermolecular hydrogen bridges are formed^21,29,30^.

## Conclusions

Both the NSP and LMA P were low-methoxylated acidic pectin, which revealed negative Q. HG was the main component of its structure. NSP of the bulb was characterized by higher branching and MW than those of foliage leaves. The MW and Z_ave,0_ of the yellow onion NSP was higher than that of the red one. The NSP was more heterogeneous in terms of particle size and had a lower amount of protein and higher TPC than LMA P. The protein content in the foliage leaves NSP was considerably higher than in the bulb NSP. The presence of proteins and phenolic compounds encourages further research into their quantitative composition and biofunctionality of onion NSP. For NSP the OHC (35–41 g/g_d.m._) was two times higher than WHC (20 g/g_d.m._), and both were higher than those for LMA P (1 and 17 g/g_d.m._, respectively). Therefore, NSP is promising material for the use in food formulations, pharmaceutic and cosmetic products (e.g. a carrier of oil-soluble active substances or hydrogel), or materials for maintaining the soil quality (e.g. oil-contaminants sorption, the moisture retention). Moreover, the surface activity of NSP was the same (decrease in γ to about 62 mN/m at 2% w/v for the red onion NSP) or higher (decrease in γ to about 56 mN/m at 2% w/v for the yellow onion NSP) than that of LMA P. This opens the possibility of NSP application in emulsion formulations. However, further studies are needed.

NSP revealed a higher ability for the macromolecule self-organization, bonding, and gelling in the acidic conditions than LMA P. Considering its negative surface charge it may carry cations important for human health or plant nutrition, for example. Further research in this direction is planned.

## Materials and methods

### Isolation of the cell wall material (CWM) and sodium carbonate-soluble pectin (NSP)

The CWM was isolated from the bulbs and foliage leaves of the yellow (*Allium cepa* L. var. Wolska) and red (*Allium cepa* L. var. Karmen) onion at the 47 407 BBCH stage of growth and development^50^. The plants were obtained from a local farmer (Turobin: 50° 49’ N, 22° 44’ E; Poland). They were washed with the tap water and next with the distilled water and the air-dried under ambient conditions (20 ± 2 °C). Next, the plant material was crushed and homogenized using a laboratory blender and it was frozen (-20 ^o^C).

The CWM was isolated following the cold extraction procedure by Ng et al.^3^ with a modification. The frozen plant material was treated with cold (4^o^ C) 70% ethanol^40^, crushed using a blender, and washed several times with the 70% ethanol at the ambient temperature (20 ± 2^o^C) up to a negative result of the sulfuric acid-UV test for the sugar presence^51^. The obtained alcohol-insoluble residue was firstly washed with 96% ethanol, next – with acetone, and it was dried to the constant mass (35 °C, 24 h, a laboratory incubator, Wamed, Warsaw, Poland). Finally, the material was crushed using a ball mill, sieved (mesh 1.4 × 0.6 mm), and it was stored in an air-dry state.

The procedure of sequential extraction of polysaccharides was based on this used for onion^3^ with modifications^52,53^. The solvents in order: the ultrapure MilliQ water, and next, the 0.5 M imidazole solution (pH 7), were used to isolate the water-soluble polysaccharides and the imidazole-soluble polysaccharides, respectively. The residue was in two steps (all night at 4 °C, and 3 h at 20 °C) and treated with the 0.05 M sodium carbonate and 20 mM sodium borohydride solution. Both supernatants containing the sodium carbonate-soluble polysaccharides (NSP) were combined. The pH was adjusted to 7, and the dialysis against the water (deionized, and finally the ultrapure water) was performed (Spectra/Por 6 MWCO 1000 tubular membranes, Roth). The suspension was freeze-dried and it was stored in tightly closed containers at the ambient conditions.

Low methyl-esterified amidated pectin (LMA P; citrus peel pectin, polygalacturonic acid ≥ 65%) was purchased from Chemat (Gdansk, Poland). It was used as a reference material in the studies.

The NSP and LMA P were dried for 24 h at 35 °C before the analyses.

### Ethics declarations

All methods were in accordance with the International Union for Conservation of Nature Policy Statement on Research Involving Species at Risk of Extinction and the Convention on International Trade in Endangered Species of Wild Fauna and Flora.

### Determination of monosaccharide and uronic acid composition of NSP

To determine the monosaccharides content in the polysaccharides the methodology by Lv et al.^54^ and Zhang et al.^55^ with some modifications was applied. Firstly, the methanolysis (2 M HCl in methanol, 80 °C, 72 h), and then hydrolysis (3 M trifluoroacetic acid (TFA), 100 °C, 7 h) were performed. Next, 1 ml of water, 50 µL of 0.3 M NaOH, and 50 µl of 0.5 M 1-phenyl-3- methyl-5-pyrazolone (PMP) in methanol were added to the residue after TFA evaporation. The mixture was conditioned at 70 °C (60 min), cooled, and neutralized (50 µl of 0.3 M HCl). Finally, the chloroform extract was obtained and filtered (22-µm). Measurements were performed using HPLC-PDA system (Sykam, Germany) and Zorbax Eclipse XDB-C18 (4.6 mm i.d. × 250 mm, 5 μm) column coupled with an Agilent Eclipse XDB-C18 guard column (4.6 mm i.d. × 12.5, 5 μm) at 30 °C. The mobile phase was: (A) 0.1 M phosphate buffer (pH 6.7) and (B) 50% v/v solution of 0.1 M phosphate buffer in acetonitrile (A:B = 69:31% (v/v)) under isocratic elution mode (the eluent flow rate 1.8 ml/min, wavelength 246 nm). Analyses were done in triplicate. The mol % of a particular component of the sample was calculated.

### Collection of the FT-IR spectra

A Nicolet 6700 FT-IR (Thermo Scientific, Madison, WI, USA) spectrometer with the Smart iTR ATR sampling accessory was used to collect the spectra over the range 4000–650 cm^−1^ (a resolution of 4 cm^−1^). For each material, three samples under the same conditions were tested and 200 scans were averaged for each spectrum. A final average spectrum was calculated and baseline correction was performed. Then, the spectra were normalized to 1.0 by dividing by surface area underneath the spectra. All the spectra manipulation was performed with OMNIC Software (Thermo Scientific).

The absorbance at the wavelength of 1600, 1681, and 1740 cm^−1^ was used for the calculation of methyl-esterification degree (DM) and the amidation degree (DA)^37,56^.

### Determination of the proteins soluble in NaCl solution

The 1 ml of 0.9% NaCl solution was added to 20 mg of the material sample. Next, it was vortexed (3000 rpm, 60 s), sonicated in the ice (3 × 10 min) and centrifuged (3000 g). The 50 µl of supernatant was taken and mixed with the cold ethanol (150 µl). Next, the samples were placed in a refrigerator (24 h). Then, they were centrifuged. The supernatant was removed and the next portion of cold ethanol was added to precipitate the protein. After 24 h of storage in a refrigerator, supernatant was removed and the precipitate was re-dissolved in 1 ml of NaCl solution. The Bradford reagent (1 ml) was added to 1 ml of sample. After 10 min the absorbance was measured (Cary 60 UV-Vis, Agilent Technologies, Inc., Santa Clara, CA, USA). The protein concentration was determined on the base of a calibration curve that was prepared using Protein Assay Kit (Thermo Scientific, Waltham, USA). For each material the analysis was performed in triplicate.

### Determination of total phenolic content (TPC)

The series of gallic acid solutions in 80% ethanol (0–0.5 mg/ml) was prepared. The 1 ml of 80% ethanol was added to 50 mg of the material sample. It was vortexed (3000 rpm, 60 s), sonicated (45 s), mixed using a rotator (1.5 h, 4 ^o^C), and finally filtered. The filtrate and gallic acid solution (0.025 mg/ml) were mixed 1:1 v/v. The 20 µl of mixture (or gallic acid solution, in a case of calibration), 1.58 ml of water, and 0.1 ml of Folin-Ciocalteau reagent were mixed, followed by 0.3 ml 20% (w/v) sodium carbonate addition. After 2 h in the dark at room temperature, the absorbance was measured at the range of 420–800 nm^57^ with the peak maximum around 756 nm^58^ (Cary 60 UV-Vis, Agilent Technologies, Inc., Santa Clara, CA, USA). The TPC was expressed as the amount of mg of gallic acid per 1 g of dry mass of polysaccharide.

### Determination of oil- (OHC) and water-holding capacity (WHC)

The Gan’ et al.^59^ procedure with modification was applied to determine the OHC and WHC of CWM. The air-dry CWM (5 mg) was put into the Eppendorf centrifuge tube and it was vortexed (3000 rpm, 60 s) with 150 µl of oil (caprylic/capric triglyceride, IBL Ltd., London, UK) or ultrapure MilliQ water. Then, samples were centrifuged (3000 rpm; 15 min.) and the supernatant was removed through decantation (a filter membrane was used to avoid the solid phase loss; the sample of liquid without the solid phase was a control). The residue was weighed. The experiment was performed in triplicate. Results were expressed as number of grams of liquid retained per 1 g of the dry mass of the solid phase.

### The light scattering analysis of the polysaccharide aqueous dispersions

The series of aqueous dispersion of pectin at the concentration ranging from 0.01 to 2% w/v were prepared by dilution of stock solution (2% w/v) with the ultrapure MilliQ water. Samples were conditioned and mixed continuously (a laboratory rotator) before the analysis for 24 h at the temperature of 20 ± 2 ^o^C. The back dynamic light scattering and laser Doppler electrophoresis measurement (minimum 12 sub-runs) were done at 20 ^o^C to obtain the values of the mean hydrodynamic diameter (Z_ave_), polydispersity index (PdI) and electrophoretic mobility (EM). Simultaneously the electrolytic conductivity (EC) was determined (conductometric method). At the concentration range of 0.01–0.08% w/v the static light scattering was measured to determine the weight–averaged molecular mass (MW) and the second virial coefficient (A_2_) as it was in^31^. The surface electrical charge of dispersed polysaccharide particles (Q) was calculated using its relationship with the EM and Z_ave_^29^. For the diluted systems (the concentration lower than 0.1% w/v), the linear relationships (linear regression, Statistica 13.1 software, StatSoft Poland Ltd., Cracow, Poland) between the Z_ave_, PdI, Q, and the polysaccharide concentration were determined. The extrapolation of the value of a given parameter to its value at the extremely diluted sample (i.e. at the concentration tending to 0) was used to obtain values of Z_ave,0_, PdI_0_, and Q_0_, that should characterize the macromolecules of polysaccharides. The light scattering measurements were carried out using the Zetasizer Nano ZS apparatus (Malvern Ltd., Malvern, UK). The analyses were done in triplicate for each material.

### The measurement of viscosity

The viscosity of the aqueous dispersions of pectin was measured at a shear rate of 200 s^−1^ using a Discovery Hybrid Rheometer (HR-1) with a parallel plate (d = 20 mm) geometry and the Peltier plate (TA Instruments, New Castle, PA, USA) at the temperature of 20 °C. It was performed in 3 repetitions.

### The measurement of pH

The Oakton Waterproof pH Spear Tester (Osprey Scientific Inc., Edmonton, AB, Canada) was used to measure pH (± 0.01) of the pectin aqueous dispersions at 20 ± 1 °C in 3 repetitions.

### The measurement of surface tension

The shape profile analysis of a pendant drop (6 µl) of the ultrapure MilliQ water and the aqueous dispersions of pectin, which gives the information on the surface tension (γ)^60^, was performed at the temperature of 20 ± 1 °C, using ramé–hart 200-U1 Goniometer/Tensiometer Instrument and the DROPimage Advanced Software (ramé–hart instrument co., Succasunna, New Jersey, USA). It was done in triplicate for each sample.

### Statistical analyses

The basic statistics, the ANOVA with post-hoc tests, and the analysis of correlations were performed for the raw data using the Statistica 13.1 software (StatSoft Polska Sp. z o.o., Cracow, Poland).

## Electronic Supplementary Material

Below is the link to the electronic supplementary material.

Supplementary Material 1

## Data Availability

This research was funded in whole or in part by National Science Centre, Poland [Grant number 2021/43/O/NZ9/02382]. For the purpose of Open Access, the author has applied a CC-BY public copyright licence to any Author Accepted Manuscript (AAM) version arising from this submission. The datasets generated and /or analysed during the current study are available from the corresponding author on reasonable request. At the time of publishing this article on the Scientific Reports website, all those data will be placed in a public open access repository.
