# Supplementary material for: Functional, physicochemical properties of sodium carbonate-soluble polysaccharides from the bulbs and foliage leaves of yellow and red onion
Source: Sci Rep. 2024 Nov 1;14:26349. doi: 10.1038/s41598-024-77936-z (PMC11530547; doi:10.1038/s41598-024-77936-z)
Supplement: Supplementary file 1 — Supplementary Material 1 [file 41598_2024_77936_MOESM1_ESM.pdf]

Functional physicochemical properties of sodium carbonate-soluble polysaccharides from the bulbs and foliage leaves of yellow and red onion.

SUPPLEMENTARY MATERIAL

Magdalena Marciniak, Jolanta Cieśla*, Monika Szymańska-Chargot,
Justyna Cybulska, Artur Zdunek

Institute of Agrophysics, Polish Academy of Sciences, Doświadczalna 4, 20-290 Lublin, Poland

Corresponding author e-mail: j.ciesla@ipan.lublin.pl

Table S1. Correlation coefficients between the parameters (raw data) describing the properties of NSPs O.

	HG	UA	Rha /UA	UA/ (Gal+Ara)	(Gal+Ara) /Rha	MW	DM	$Z_{ave,0}$	PdI_0	Q_0	NaClS protein	TPC	OHC	WHC
UA	**0.99**													
Rha/UA	**-0.83**	**-0.78**												
UA/ (Gal+Ara)	**0.94**	**0.94**	**-0.69**											
(Gal+Ara)/ Rha	**-0.69**	**-0.74**	0.26	**-0.82**										
MW	-0.34	-0.32	0.41	-0.42	0.32									
DM	-0.34	-0.35	0.19	-0.34	0.35	-0.28								
$Z_{ave,0}$	-0.45	-0.42	0.34	**-0.69**	0.53	0.47	0.12							
PdI_0	0.50	0.53	-0.19	0.49	-0.47	0.27	-0.45	-0.05						
Q_0	0.19	0.20	-0.22	0.10	-0.19	0.18	-0.05	0.11	0.24					
NaClS protein	0.40	0.37	-0.43	0.55	-0.41	**-0.92**	0.15	**-0.75**	-0.29	-0.21				
TPC	**0.79**	**0.79**	-0.57	**0.83**	**-0.68**	-0.40	-0.39	-0.48	**0.69**	0.15	0.42			
OHC	-0.38	-0.39	0.28	-0.52	0.53	**0.64**	-0.34	0.53	0.15	-0.01	**-0.69**	-0.26		
WHC	0.25	0.25	-0.22	0.07	0.07	**0.68**	-0.47	0.24	0.36	0.17	**-0.58**	0.01	0.53	
$\gamma_{2\%}$	**0.60**	**0.62**	-0.31	**0.77**	**-0.70**	-0.48	-0.0819	**-0.67**	0.42	-0.25	**0.59**	**0.72**	**-0.66**	-0.32

NSPs O – sodium carbonate – soluble pectic polysaccharides of onion, HG – the content (mol %) of homogalacturonan, UA – the content (mol %) of uronic acids, Rha/UA – the ratio of the rhamnose (mol %) to the uronic acids content (mol %), UA/(Gal+Ara) – the ratio of the uronic acids content (mol %) to the sum of galactose (mol %) and arabinose content (mol %), (Gal+Ara)/Rha – the ratio of the sum of galactose (mol %) and arabinose content (mol %) to the rhamnose content (mol %), MW – the weight average molecular mass (kDa), DM – the methyl esterification degree (%), $Z_{ave,0}$ – the mean hydrodynamic diameter (nm) at the extreme dilution of the polysaccharide aqueous dispersion, PdI_0 – polydispersity index of the extremely diluted polysaccharide aqueous dispersion, Q_0 – the net surface electrical charge of macromolecule (mC/m^2), NaClS protein – the content of salt soluble protein ($mg/g_{d.m.}$), TPC – the total phenolic content ($mg/g_{d.m.}$), OHC – the oil holding capacity ($g/g_{d.m.}$), WHC – the water holding capacity ($g/g_{d.m.}$), $\gamma_{2\%}$ – the surface tension (mN/m) of NSP aqueous dispersion at the concentration of 2 % w/v; the red font on yellow background means the statistically significant correlation ($p < 0.05$).

Table S2. The results of three-way ANOVA and post-hoc HSD Tukey' test at $p < 0.05$ for the effects of the source of pectin (YO, RO, LMA P), organ (B and L), and the concentration of aqueous dispersion on the surface tension (raw data).

Concentration (m/v %)	The sample				
	NSP YO B	NSP YO L	NSP RO B	NSP RO L	LMA P
0.01	a	ab	a	ab	ab
0.05	a	ab	ab	ab	ab
0.10	a	ab	a	ab	ab
0.25	a	ab	a	b	ab
0.50	ab	bc	ab	bc	bc
1.00	bc	d	cd	cd	cd
2.00	g	g	f	ef	e

NSP YO B – sodium carbonate – soluble pectic polysaccharide (NSP) of yellow (Y) onion (O) bulb (B), NSP YO L – sodium carbonate – soluble pectic polysaccharide (NSP) of yellow (Y) onion (O) foliage leaves (L), NSP RO B – sodium carbonate – soluble pectic polysaccharide (NSP) of red (R) onion (O) bulb (B), NSP RO L – sodium carbonate – soluble pectic polysaccharide (NSP) of red (R) onion (O) foliage leaves (L), and LMA P – low-methoxylated amidated pectin from citrus peel; the same letters mean not significant differences between the results; the cells marked in colour point out the concentration above which there was a significant decrease in a surface tension.

Table S3. The results of three-way ANOVA and post-hoc HSD Tukey' test at $p < 0.05$ for the effects of the source of pectin (YO, RO, LMA P), organ (B and L), and the concentration of aqueous dispersion on the Relative Z_{ave} (raw data).

Concentration (m/v %)	The sample NSP YO B	NSP YO L	NSP RO B	NSP RO L	LMA P
0.01	kl	l	l	l	l
0.02	kl	kl	jkl	l	l
0.03	jkl	kl	kl	l	l
0.04	jkl	kl	kl	kl	kl
0.05	jkl	jkl	jkl	l	kl
0.06	jkl	jkl	jkl	kl	kl
0.07	jkl	jkl	jkl	kl	kl
0.08	jkl	jkl	jkl	kl	kl
0.125	jkl	jkl	jkl	kl	kl
0.25	hi	ijk	hij	jkl	jkl
0.50	de	f	g	jkl	jkl
1.00	a	cd	e	g	jkl
2.00	bc	a	ghi	b	gh

NSP YO B – sodium carbonate – soluble pectic polysaccharide (NSP) of yellow (Y) onion (O) bulb (B), NSP YO L – sodium carbonate – soluble pectic polysaccharide (NSP) of yellow (Y) onion (O) foliage leaves (L), NSP RO B – sodium carbonate – soluble pectic polysaccharide (NSP) of red (R) onion (O) bulb (B), NSP RO L – sodium carbonate – soluble pectic polysaccharide (NSP) of red (R) onion (O) foliage leaves (L), and LMA P – low-methoxylated amidated pectin from citrus peel; the same letters mean not significant differences between the results; the cells marked in colour point out the concentration above which there was a significant increase in Relative Z_{ave}.

Table S4. The results of three-way ANOVA and post-hoc HSD Tukey' test at $p < 0.05$ for the effects of the source of pectin (YO, RO, LMA P), organ (B and L), and the concentration of aqueous dispersion on the Polydispersity Index (PdI) raw data.

Concentration (m/v %)	The sample NSP YO B	NSP YO L	NSP RO B	NSP RO L	LMA P
0.01	bc	cd	bc	cd	f
0.02	bc	cd	abc	de	f
0.03	cd	d	e	e	f
0.04	cd	d	cd	cd	f
0.05	d	de	de	de	f
0.06	d	de	d	e	f
0.07	d	de	d	de	f
0.08	de	de	abc	d	f
0.125	de	de	d	d	f
0.25	de	de	bc	ab	f
0.50	e	e	a	a	f
1.00	e	e	de	ab	ef
2.00	f	e	a	de	ef

NSP YO B – sodium carbonate – soluble pectic polysaccharide (NSP) of yellow (Y) onion (O) bulb (B), NSP YO L – sodium carbonate – soluble pectic polysaccharide (NSP) of yellow (Y) onion (O) foliage leaves (L), NSP RO B – sodium carbonate – soluble pectic polysaccharide (NSP) of red (R) onion (O) bulb (B), NSP RO L – sodium carbonate – soluble pectic polysaccharide (NSP) of red (R) onion (O) foliage leaves (L), and LMA P – low-methoxylated amidated pectin from citrus peel; the same letters mean not significant differences between the results.

Table S5. The results of three-way ANOVA and post-hoc HSD Tukey' test at $p < 0.05$ for the effects of the source of pectin (YO, RO, LMA P), organ (B and L), and the concentration of aqueous dispersion on the viscosity (raw data).

Concentration (m/v %)	The sample				
	NSP YO B	NSP YO L	NSP RO B	NSP RO L	LMA P
0.01	h	h	h	h	h
0.05	h	h	h	h	h
0.10	h	h	h	h	h
0.25	h	h	h	h	h
0.50	g	g	g	h	h
1.00	e	f	e	g	h
2.00	a	c	b	d	g

NSP YO B – sodium carbonate – soluble pectic polysaccharide (NSP) of yellow (Y) onion (O) bulb (B), NSP YO L – sodium carbonate – soluble pectic polysaccharide (NSP) of yellow (Y) onion (O) foliage leaves (L), NSP RO B – sodium carbonate – soluble pectic polysaccharide (NSP) of red (R) onion (O) bulb (B), NSP RO L – sodium carbonate – soluble pectic polysaccharide (NSP) of red (R) onion (O) foliage leaves (L), and LMA P – low-methoxylated amidated pectin from citrus peel; the same letters mean not significant differences between the results; the cells marked in colour point out the concentration above which there was a significant increase in viscosity.

Table S6. The results of three-way ANOVA and post-hoc HSD Tukey' test at $p < 0.05$ for the effects of the source of pectin (YO, RO, LMA P), organ (B and L), and the concentration of aqueous dispersion on the electrolytic conductivity (raw data).

Concentration (m/v %)	The sample				
	NSP YO B	NSP YO L	NSP RO B	NSP RO L	LMA P
0.01	n	n	n	n	n
0.02	mn	mn	mn	mn	n
0.03	mn	mn	mn	mn	mn
0.04	mn	mn	mn	mn	mn
0.05	mn	mn	mn	mn	mn
0.06	mn	mn	mn	mn	mn
0.07	mn	mn	mn	mn	mn
0.08	mn	mn	mn	mn	mn
0.125	mn	lm	lm	l	mn
0.25	kl	kl	k	jk	l
0.50	ij	hi	hi	g	k
1.00	f	f	e	d	gh
2.00	c	bc	b	a	e

NSP YO B – sodium carbonate – soluble pectic polysaccharide (NSP) of yellow (Y) onion (O) bulb (B), NSP YO L – sodium carbonate – soluble pectic polysaccharide (NSP) of yellow (Y) onion (O) foliage leaves (L), NSP RO B – sodium carbonate – soluble pectic polysaccharide (NSP) of red (R) onion (O) bulb (B), NSP RO L – sodium carbonate – soluble pectic polysaccharide (NSP) of red (R) onion (O) foliage leaves (L), and LMA P – low-methoxylated amidated pectin from citrus peel; The same letters mean not significant differences between the results; the cells marked in colour point out the concentration above which there was a significant increase in electrolytic conductivity.

Table S7. The results of three-way ANOVA and post-hoc HSD Tukey' test at $p < 0.05$ for the effects of the source of pectin (YO, RO, LMA P), organ (B and L), and the concentration of aqueous dispersion on the pH (raw data).

Concentration (m/v %)	The sample				
	NSP YO B	NSP YO L	NSP RO B	NSP RO L	LMA P
0.01	c	kl	c	ef	a
0.02	d	m	e	gh	b
0.03	ef	mn	fg	hi	de
0.04	h	n	hi	i	fg
0.05	h	mn	i	i	h
0.06	i	o	j	jk	i
0.07	i	o	ij	jk	i
0.08	jk	o	kl	kl	i
0.125	lm	op	kl	l	jk
0.25	o	pr	n	n	m
0.50	op	r	op	pr	no
1.00	pr	s	p	r	s
2.00	pr	t	p	s	t

NSP YO B – sodium carbonate – soluble pectic polysaccharide (NSP) of yellow (Y) onion (O) bulb (B), NSP YO L – sodium carbonate – soluble pectic polysaccharide (NSP) of yellow (Y) onion (O) foliage leaves (L), NSP RO B – sodium carbonate – soluble pectic polysaccharide (NSP) of red (R) onion (O) bulb (B), NSP RO L – sodium carbonate – soluble pectic polysaccharide (NSP) of red (R) onion (O) foliage leaves (L), and LMA P – low-methoxylated amidated pectin from citrus peel; the same letters mean not significant differences between the results.

Table S8. The results of three-way ANOVA and post-hoc HSD Tukey' test at $p < 0.05$ for the effects of the source of pectin (YO, RO, LMA P), organ (B and L), and the concentration of aqueous dispersion on the surface electrical charge (raw data).

Concentration (m/v %)	The sample				
	NSP YO B	NSP YO L	NSP RO B	NSP RO L	LMA P
0.01	a	a	a	a	a
0.02	b	bc	c	a	b
0.03	d	c	bc	a	a
0.04	d	c	b	b	a
0.05	e	cd	cd	a	ab
0.06	ef	d	d	c	b
0.07	f	d	e	bc	b
0.08	f	de	e	c	b
0.125	g	ef	e	c	bc
0.25	gh	g	g	f	d
0.50	h	h	h	fg	ef
1.00	h	h	h	h	g
2.00	h	h	h	h	h

NSP YO B – sodium carbonate – soluble pectic polysaccharide (NSP) of yellow (Y) onion (O) bulb (B), NSP YO L – sodium carbonate – soluble pectic polysaccharide (NSP) of yellow (Y) onion (O) foliage leaves (L), NSP RO B – sodium carbonate – soluble pectic polysaccharide (NSP) of red (R) onion (O) bulb (B), NSP RO L – sodium carbonate – soluble pectic polysaccharide (NSP) of red (R) onion (O) foliage leaves (L), and LMA P – low-methoxylated amidated pectin from citrus peel; the same letters mean not significant differences between the results.

Table S9. Correlation coefficients between the parameters (raw data) describing the NSPs O and their aqueous dispersions.

	$RZ_{ave\ C}$	$RZ_{ave,1\%}$	$PdI_{1\%}$	$Viscosicy_{C}$	$Viscosity_{1\%}$	$EC_{1\%}$	$Q_{1\%}$
HG	0.60	-0.73	0.61	0.34	-0.34	0.65	-0.68
UA	0.58	-0.71	0.58	0.30	-0.32	0.63	-0.65
Rha/UA	-0.55	0.63	-0.61	-0.38	0.45	-0.50	0.63
UA/ (Gal+Ara)	0.75	-0.86	0.72	0.52	-0.41	0.81	-0.79
(Gal+Ara)/ Rha	-0.60	0.69	-0.53	-0.35	0.31	-0.64	0.53
MW	-0.87	0.72	-0.86	-0.88	0.95	-0.68	0.749
$Z_{ave,0}$	-0.80	0.84	-0.74	-0.78	0.49	-0.86	0.829
NaClS perotein	0.96	-0.87	0.92	0.98	-0.91	0.82	-0.89
TPC	0.58	-0.64	0.66	0.43	-0.23	0.74	-0.62
$RZ_{ave\ c}$		-0.95	0.97	0.93	-0.87	0.90	-0.93
$RZ_{ave,1\%}$			-0.90	-0.82	0.75	-0.88	0.95
$PdI_{\ 1\%}$				0.89	-0.84	0.91	-0.90
$Viscosity_{C}$					-0.84	0.84	-0.87
$Viscosity_{\ 1\%}$						-0.61	0.74
$EC_{\ 1\%}$							-0.92

NSPs O – sodium carbonate – soluble pectic polysaccharide of onion, HG – the content (mol %) of homogalacturonan, UA – the content (mol %) of uronic acids, Rha/UA – the rhamnose to uronic acids mol % ratio, UA/(Gal+Ara) – the ratio of the uronic acids content (mol %) to the sum of galactose (mol %) and arabinose content (mol %), (Gal+Ara)/Rha – the ratio of the sum of galactose (mol %) and arabinose content (mol %) to the rhamnose content (mol %), MW – the weight average molecular mass (kDa), DM – the methyl esterification degree (%), $Z_{ave,0}$ – the mean hydrodynamic diameter (nm) at the extreme dilution of the polysaccharide aqueous dispersion, NaClS protein – the content of salt soluble protein (mg/$g_{d.m.}$), TPC – the total phenolic content (mg/$g_{d.m.}$), $RZ_{ave\ C}$ – the concentration of aqueous dispersion of NSP above which a significant increase in the Relative mean hydrodynamic diameter occurred, $RZ_{ave,1\%}$ – the value of Relative mean hydrodynamic diameter at the concentration of 1 % w/v of aqueous NSP dispersion, $PdI_{1\%}$ – polydispersity index of the 1 % w/v aqueous NSP dispersion, $Viscosicy_{C}$ - the concentration of aqueous dispersion of NSP above which a significant increase in viscosity occurred, $Viscosity_{1\%\ \%}$ – the value of viscosity of the 1 % w/v aqueous NSP dispersion, $EC_{1\%\ \%}$ – the value of electrolytic conductivity of the 1 % w/v aqueous NSP dispersion, $Q_{1\%\ \%}$ – the value of net surface electrical charge of particles in the 1 % w/v aqueous NSP dispersion; the parameters analysed in Table S1 were omitted in Table S9; the red font on yellow background means the statistically significant correlation ($p < 0.05$).
